# Antiviral activity of carnosic acid against respiratory syncytial virus

**DOI:** 10.1186/1743-422X-10-303

**Published:** 2013-10-08

**Authors:** Han-Bo Shin, Myung-Soo Choi, Byeol Ryu, Na-Rae Lee, Hye-In Kim, Hye-Eun Choi, Jun Chang, Kyung-Tae Lee, Dae Sik Jang, Kyung-Soo Inn

**Affiliations:** 1 Department of Pharmaceutical Science, College of Pharmacy, Kyung Hee University, College of Pharmacy, 26 Kyungheedae-ro, Dongdaemun-gu, Seoul 130-701, Korea; 2 Department of Pharmaceutical Biochemistry and Department of Life and Nanopharmaceutical Science, College of Pharmacy, Kyung Hee University, 26 Kyungheedae-ro, Dongdaemun-gu, Seoul 130-701, Korea; 3 College of Pharmacy, Ewha Womans University, 52, Ewhayeodae-gil, Seodaemun-gu, Seoul 120-750, Korea

**Keywords:** hRSV, *Rosemarinus officinalis*, Carnosic acid, Anti-hRSV activity

## Abstract

**Background:**

Human respiratory syncytial virus (hRSV) is a leading cause of severe lower respiratory infection and a major public health threat worldwide. To date, no vaccine or effective therapeutic agent has been developed. In a screen for potential therapeutic agents against hRSV, we discovered that an extract of *Rosmarinus officinalis* exerted a strong inhibitory effect against hRSV infection. Subsequent studies identified carnosic acid as a bioactive constituent responsible for anti-hRSV activity. Carnosic acid has been shown to exhibit potent antioxidant and anti-cancer activities. Anti-RSV activity of carnosic acid was further investigated in this study.

**Methods:**

Effects of extracts from various plants and subfractions from *R. officinalis* on hRSV replication were determined by microneutralization assay and plaque assay. Several constituents were isolated from ethyl acetate fraction of *R. officinalis* and their anti-RSV activities were assessed by plaque assay as well as reverse-transcription quantitative PCR to determine the synthesis of viral RNAs.

**Results:**

Among the tested bioactive constituents of *R. officinalis*, carnosic acid displayed the most potent anti-hRSV activity and was effective against both A- and B-type viruses. Carnosic acid efficiently suppressed the replication of hRSV in a concentration-dependent manner. Carnosic acid effectively suppressed viral gene expression without inducing type-I interferon production or affecting cell viability, suggesting that it may directly affect viral factors. A time course analysis showed that addition of carnosic acid 8 hours after infection still effectively blocked the expression of hRSV genes, further suggesting that carnosic acid directly inhibited the replication of hRSV.

**Conclusions:**

The current study demonstrates that carnosic acid, a natural compound that has already been shown to be safe for human consumption, has anti-viral activity against hRSV, efficiently blocking the replication of this virus. Carnosic acid inhibited both A- and B- type hRSV, while it did not affect the replication of influenza A virus, suggesting that its antiviral activity is hRSV-specific. Collectively, this study suggests the need for further evaluation of carnosic acid as a potential treatment for hRSV.

## Background

Human respiratory syncytial virus (hRSV), a single-stranded RNA virus of the family Paramyxoviridae, is a leading cause of lower respiratory tract infection in infants and children [1]. This is especially true for high-risk groups, including infants with congenital heart disease and immunosuppressed patients, where infection by hRSV causes severe mortality [2]. It has been estimated that acute lower respiratory infection by hRSV caused approximately 66,000–199,000 deaths of children under the age of five worldwide in 2005 [3]. Despite the severity of the global health threat and economic burden posed by hRSV infection, no effective hRSV-specific antiviral agents have been developed to date. Ribavirin, a nucleoside analog, is the only approved drug for severe hRSV infection, but it is not recommended owing to several factors, including its toxicity and difficulty of administration [4]. Notably, ribavirin has shown limited efficacy in clinical trials on children with bronchiolitis caused by hRSV [5-8]. Although immunoprophylaxis with an hRSV-neutralizing antibody (palivizumab, MedImmune) confers substantial protection against severe hRSV diseases, no licensed vaccine is currently available. Thus, there is an urgent need for the development of effective and safe vaccines and therapeutics. Previous studies showed inhibition of hRSV by several natural compounds, including amentoflavone [9]; anagyrine, oxymatrine, sophoranol, wogonin, and oroxylin A [10]; 3-alpha-hydroxy-lup-20(2 9)-ene-23,28-dioic acid and 3-epi-betulinic acid 3-O-sulfate [11]; and cimicifugin [12].

Extract from *Rosmarinus officinalis* (Rosemary) has been known to possess strong antioxidant activity and widely used for food preservation [13]. In traditional medicine, *R. officinalis* has been used to treat various conditions [14]. In addition, *R. officinalis* has been used as a traditional medicine to treat various infectious diseases, and its antimicrobial effect against various bateria has been proved by several studies [15-17]. Among its constituents, carnosic acid (CAS #3650-09-7), a benzenediol abietane diterpene and its degradation product carnosol are well known antioxidative compounds [18]. In addition to its antioxidant activity, carnosic acid exerts growth-inhibitory effects on breast cancer cells [19], ovarian cancer cells [20], and prostate cancer cells [21] by inducing apoptosis. Carnosic acid also possesses anti-bacterial, anti-inflammatory and neuroprotective activities [22-24]. Although carnosic acid has been shown to inhibit human immunodeficiency virus (HIV) protease activity [25], little is known about the antiviral actions of carnosic acid.

In this study, we found that carnosic acid from *R. officinalis* was capable of inhibiting hRSV infection and replication, suggesting its potential therapeutic and prophylactic use against hRSV infection.

## Results and discussion

### *R. Officinalis* extract inhibits the replication of hRSV

**Figure 1:**
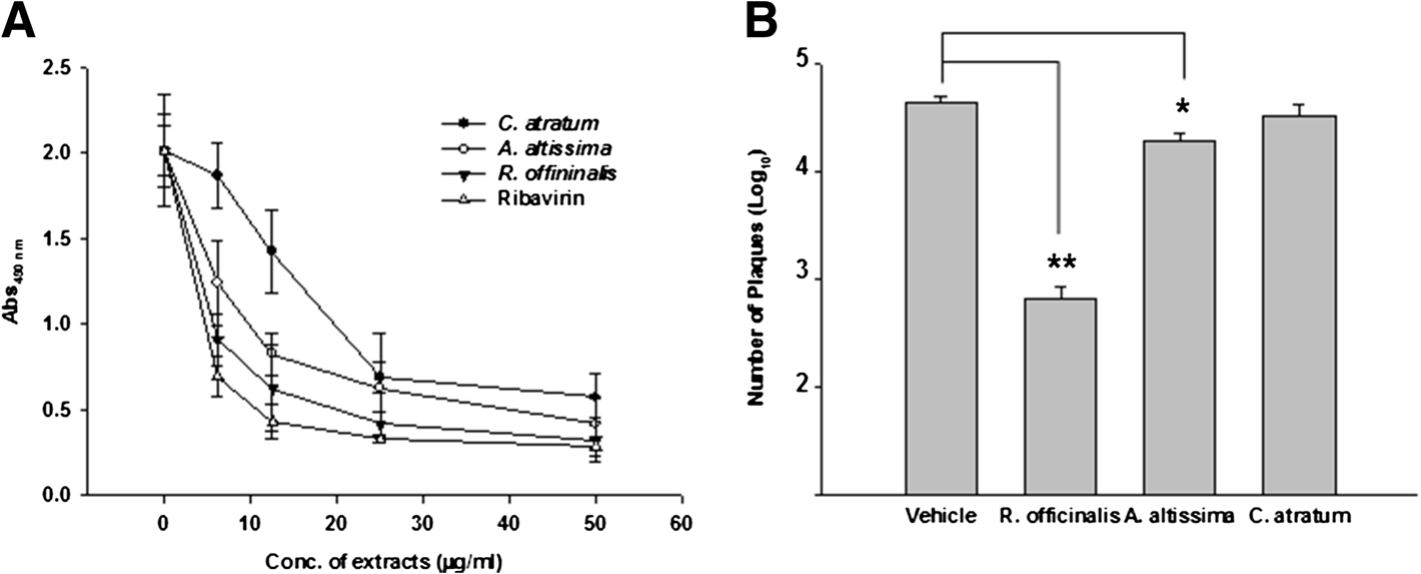
**Antiviral activity of *R. Officinalis *extract against hRSV. (A)** Anti-hRSV effects of extracts of *A. altissima*, *C. atratum*, and *R. officinalis*. A549 cells (5 **×** 10^4^ cells/well) were infected with hRSV A2 (1000 pfu) and treated with different concentrations of total extracts or vehicle (DMSO). Expression of hRSV fusion (F) protein was assessed by microneutralization assay using an anti-F antibody. The assay was performed in duplicate and data represent means ± SD. **(B)** Viral yield-reduction assay. A549 cells were treated with vehicle or 20 μg/ml of the indicated compounds 1 hour prior to hRSV infection (0.5 MOI). Viruses were harvested 3 days after infection and titrated by plaque assay. Data represent number of plaques (means ± SD). * *p* < 0.05 versus vehicle. ** *p* < 0.01 versus vehicle.

In order to identify natural antiviral agents against hRSV, we screened MeOH extracts of a variety of plants for anti-hRSV activities using a microneutralization assay. Among tested samples, extracts from *Ailanthus altissima*, *Cynanchum atratum*, and *R. officinalis* displayed concentration-dependent inhibition of the expression of F protein in hRSV-infected cells (Figure 1A). To confirm the anti-hRSV activities of the extracts, we determined their effects on the production of hRSV from infected cells. A549 cells in media containing 20 μg/ml of each extract were infected with hRSV A2 virus at an MOI (multiplicity of infection) of 0.5 for 3 days, followed by an analysis of virus production by plaque assay. As shown in Figure 1B, treatment with *R. officinalis* extract reduced virus production by approximately a 66-fold compared to vehicle treatment, whereas treatment with *A. altissima* extract resulted in approximately a 2.2-fold reduction and *C. atratum* extract had no significant effect. These results suggest that some components of *R. officinalis* possess anti-hRSV activity. Thus, *R. officinalis* extract was used in subsequent experiments designed to isolate anti-hRSV compound(s).

### Isolation of compounds from *R. officinalis*

**Figure 2:**
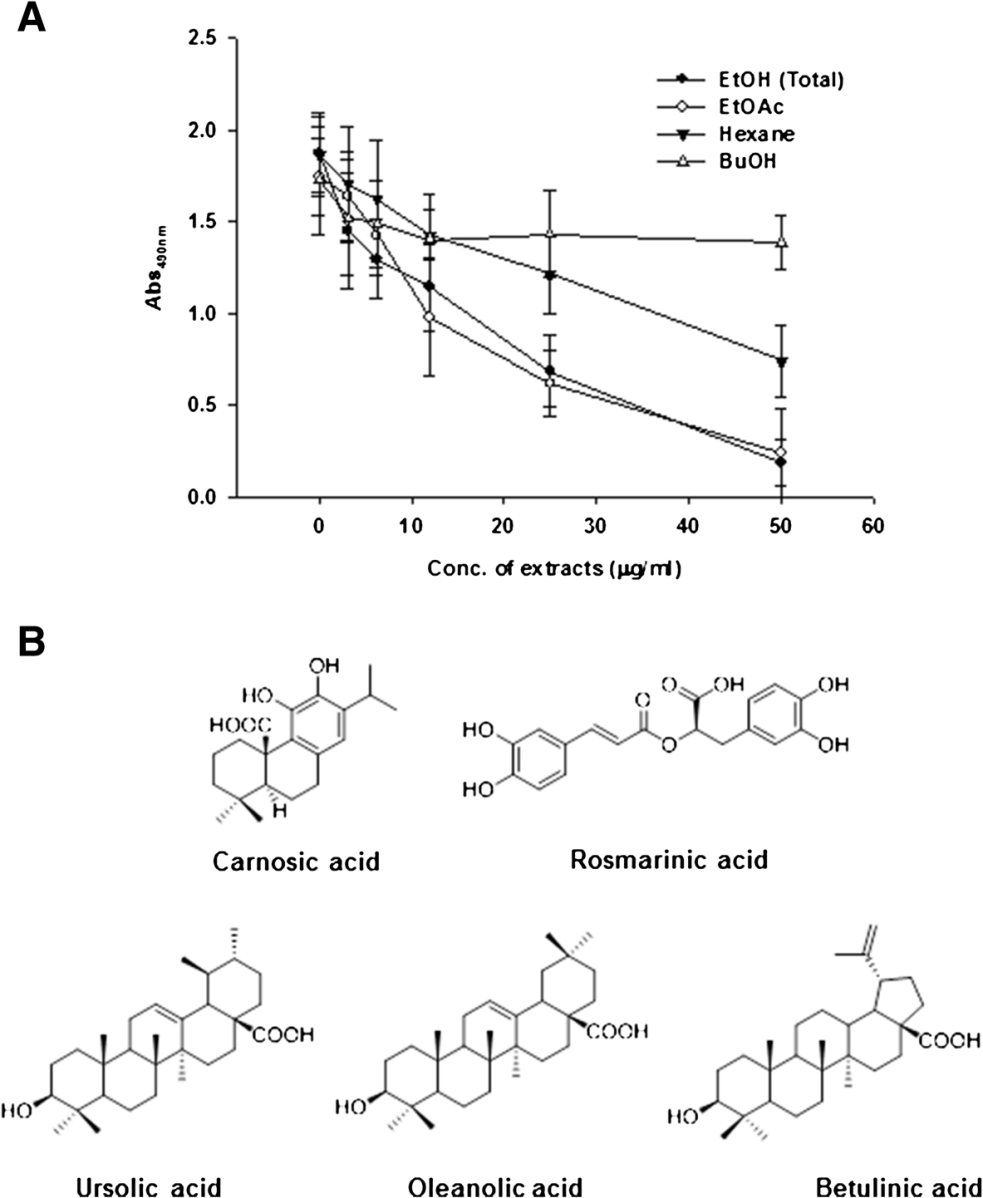
**Isolation of compounds from *R. officinalis. *(A)** Anti-hRSV effect of the EtOAc fraction of *R. officinalis* EtOH extract. Anti-hRSV activities of EtOAc-soluble, hexane-soluble, BuOH-soluble, and total EtOH extracts were assessed by microneutralization assay as described in Figure 1. Data represent means ± SD. **(B)** Chemical structures of compounds isolated from the EtOAc fraction, which showed the strongest anti-hRSV activity.

*n-*Hexane-soluble, EtOAc-soluble, BuOH-soluble, and water-soluble fractions were extracted from the total EtOH extract of *R. officinalis* as described in Materials and Methods. Fractions capable of inhibiting hRSV replication were identified by testing their effects on hRSV replication using a microneutralization assay. As depicted in Figure 2A, the EtOAc-extracted fraction displayed anti-hRSV activity comparable to that of the total EtOH extract of *R. officinalis*, suggesting the existence of hRSV-inhibiting compounds in the EtOAc faction. To identify the molecule responsible for the inhibitory activity, we further purified the EtOAc-soluble extract by column chromatography on silica gel, eluting with *n-*hexane−EtOAc−MeOH (7:3:0 ↓ 0:1:0 ↓ 0:9:1 ↓ 0:4:1 v/v; final stage, MeOH 100%) to yield seven fractions (F01−F07). Fraction F02 was chromatographed over reversed-phase silica gel (MeOH–H_2_O gradient from 4:1 to 19:1, v/v) to give carnosic acid and betulinic acid. Ursolic acid and oleanolic acid were obtained from fraction F03 by reversed-phase column chromatography, eluting with MeOH–H_2_O (19:1, v/v). Fraction F07 was chromatographed over reversed-phase silica gel to give rosmarinic acid. Chemical structures of isolated compounds are shown in Figure 2B.

### Identification of carnosic acid as the compound responsible for anti-hRSV activity

**Figure 3:**
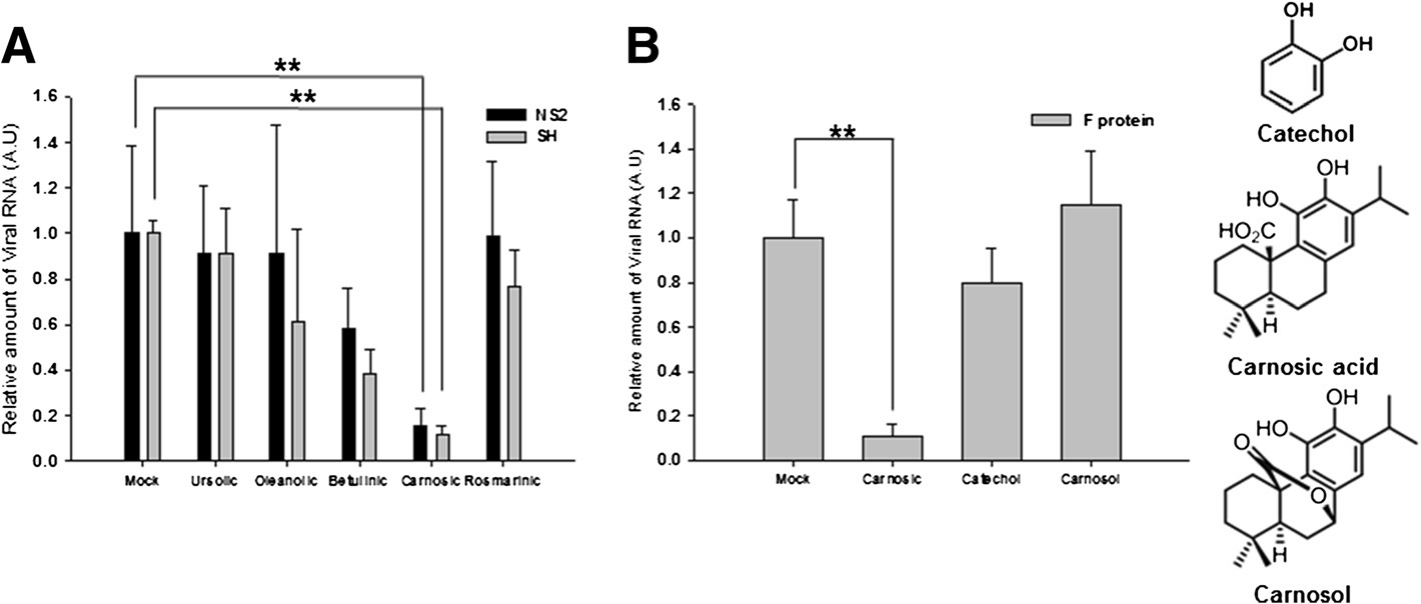
**Identification of compounds responsible for anti-hRSV activity. (A)** Inhibitory activity of isolated compounds toward viral protein synthesis. A549 cells were treated with the indicated compounds 1 hour prior to hRSV infection (MOI 0.5). cDNAs were synthesized from total RNA isolated from cells 24 hours after infection. The relative amount of RNA for hRSV NS2 and SH protein were determined by RT-qPCR. Data represent means ± SD. ** *p* < 0.01 versus Mock-treated (Mock). A.U.: Arbitrary unit. **(B)** Comparison of inhibitory activities of carnosic acid, catechol, and carnosol. Effects of carnosic acid (carnosic), catechol, and carnosol on the synthesis of hRSV F protein RNA was determined as in **(A)**. Data represent means ± SD. ** *p* < 0.01 versus Mock-treated (Mock). A.U.: Arbitrary unit.

To identify the compound capable of inhibiting hRSV replication, we assessed the anti-hRSV activities of isolated compounds by RT-qPCR using primers specific for SH and NS2 proteins of hRSV. Among tested compounds, carnosic acid exerted the strongest inhibition of viral RNA synthesis (Figure 3A). It is known that *R. officinalis* contains carnosol, which has a chemical structure very similar to that of carnosic acid. Thus, we compared the inhibitory activity of carnosic acid with that of carnosol. Interestingly, carnosol did not show any significant inhibitory activity against hRSV (Figure 3B). In addition, catechol failed to suppress hRSV RNA synthesis, suggesting that the carboxylic acid moiety of carnosic acid might be crucial for its anti-hRSV activity (Figure 3B).

### Effect of carnosic acid on interferon production upon hRSV infection

**Figure 4:**
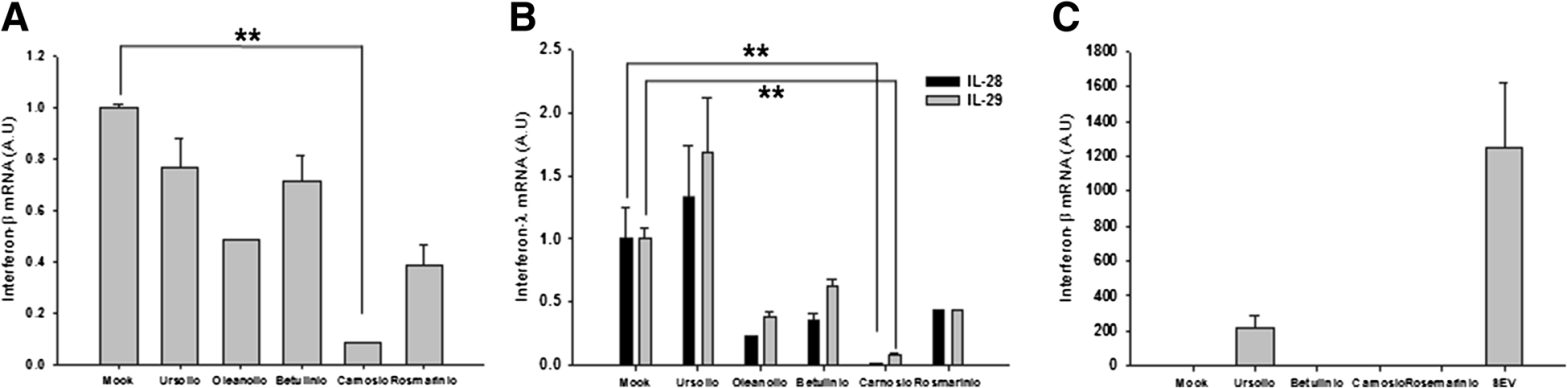
**Effects of carnosic acid on interferon production.** Effects of isolated compounds on interferon-β **(A)** and interferon-λ1 and -λ2 **(B)** production were analyzed by RT-qPCR using the same samples from Figure 3. Data represent means ± SD. ** *p* < 0.01 versus Mock-treated (Mock). A.U.: Arbitrary unit. **(C)** To determine the effect of isolated compounds themselves on interferon- β production, A549 cells were treated with carnosic acid without virus infection. Data represent means ± SD. A.U.: Arbitrary unit.

Type-I and type-III interferons play crucial role in antiviral immune responses against hRSV. hRSV antagonize the induction of interferons and their actions to escape from immune surveillance using nonstructural (NS) proteins, NS1 and NS2 [26-29]. Thus, we tested the possibility that treatment of carnosic acid induces interferons and subsequently suppresses the replication of hRSV. None of the tested compounds upregulated the production of interferon-β or interferon-λ during hRSV infection, suggesting that the inhibitory activity of carnosic acid was not due to the induction of interferon (Figure 4A, B). In fact, the production of interferon-β and interferon-λ in carnosic acid-treated cells was actually much lower than that in vehicle-treated cells after virus infection, possibly due to the low level of hRSV infection or replication. Treatment of carnosic acid without virus infection did not induce interferon- β, while Sendai virus induced high level of interferon-β mRNA (Figure 4C).

### Analysis of cytotoxic activity of carnosic acid

**Figure 5:**
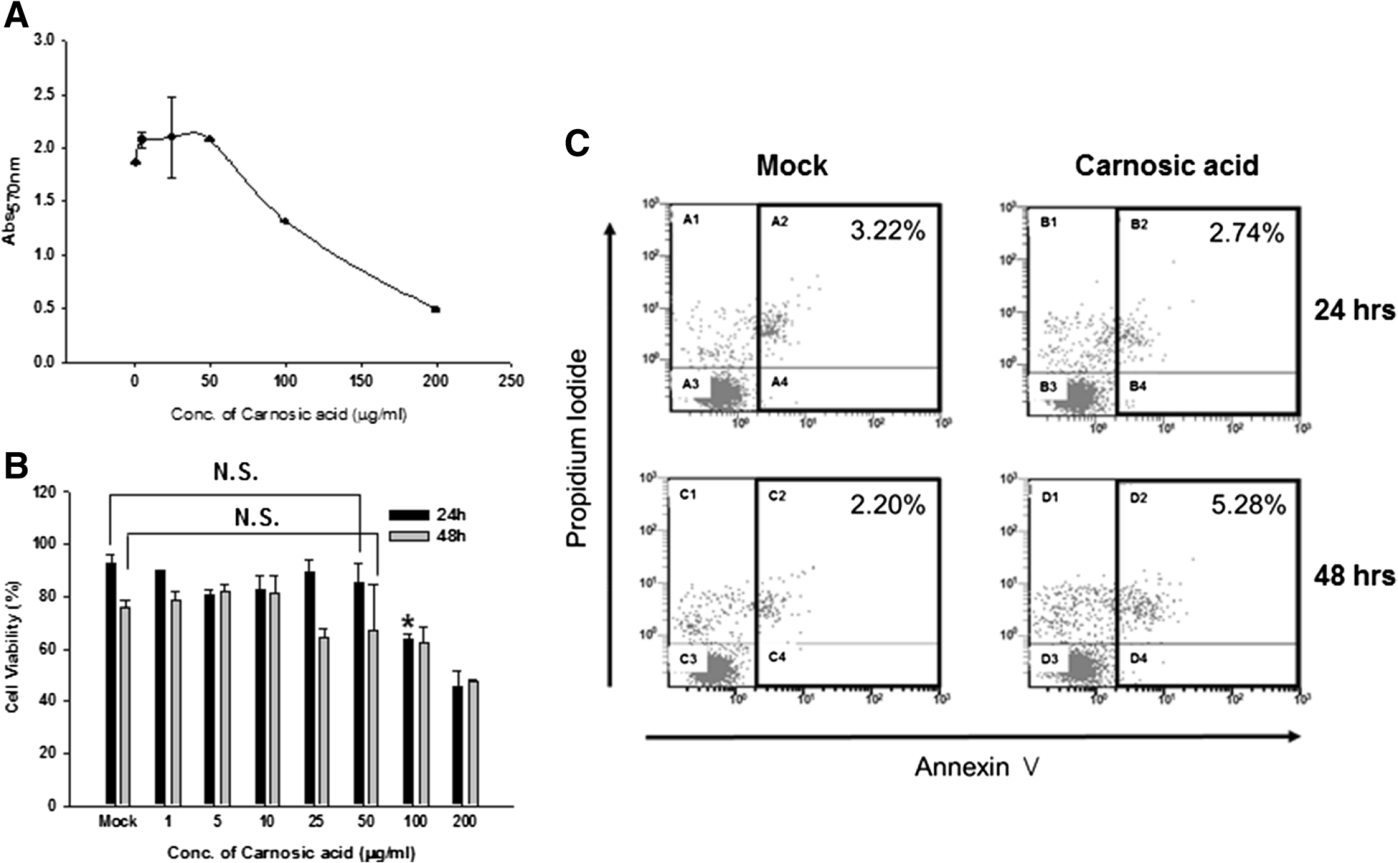
**Effects of carnosic acid on cell viability. (A)** Cell cytotoxicity assay. A549 cells were treated with increased concentration of carnosic acid for 24 hours and subjected to a MTT assay. Data represent means ± SD. **(B)** Viable cell count assay. A549 cells were treated with increased amount of carnosic acid for 24 hours or 48 hours. Cells were detached and mixed with trypan blue cell staining solution. Viable cells and dead cells were counted under microscope. All the experiments were performed in triplicate and data represent the percentage of viable cells (means ± SD). N.S.: not significant. * *p* < 0.01 versus Mock-treated (Mock). **(C)** Apoptosis assay. Apoptotic cell death of A549 cells incubated with or without carnosic acid (20 μg/ml) for 24 and 48 hours was analyzed by flow cytometry using Annexin V-FITC and propidium iodide.

To rule out the possibility that nonspecific cytotoxicity of carnosic acid could affect hRSV replication, we examined the cytotoxicity of carnosic acid by MTT assay. Carnosic acid did not show any significant cytotoxicity at the concentrations used for antiviral assays (Figure 5A). The 50% cytotoxic concentration of carnosic acid was 130.8 μg/ml. Effect of carnosic acid on cell viability was further examined by trypan blue exclusion assay and apoptosis analysis. As shown in Figure 5B, cells treated with 50 μg/ml of carnosic acid for 24 hours did not show any significant decrease in cell viability. The percentage of viable cells was slightly lowered from 75% to 67 and 62% by the treatment of 50 μg/ml and 100 μg/ml of carnosic acid for 48 hours, respectively. Similarly, the treatment of carnosic acid (20 μg/ml) for 24 hours did not induce apoptotic cell death of A549 cells. Annexin V-positive apoptotic cells were slightly increased from 2.2% to 5.28% by the treatment of carnosic acid for 48 hours (Figure 5C). Thus, we concluded that carnosic acid does not induce severe cell cytotoxicity at the concentration lower than 50 μg/ml and the antiviral activity of carnosic acid is not due to the non-specific cell cytotoxicity of carnosic acid.

### Assessment of the anti-hRSV activity of carnosic acid

**Figure 6:**
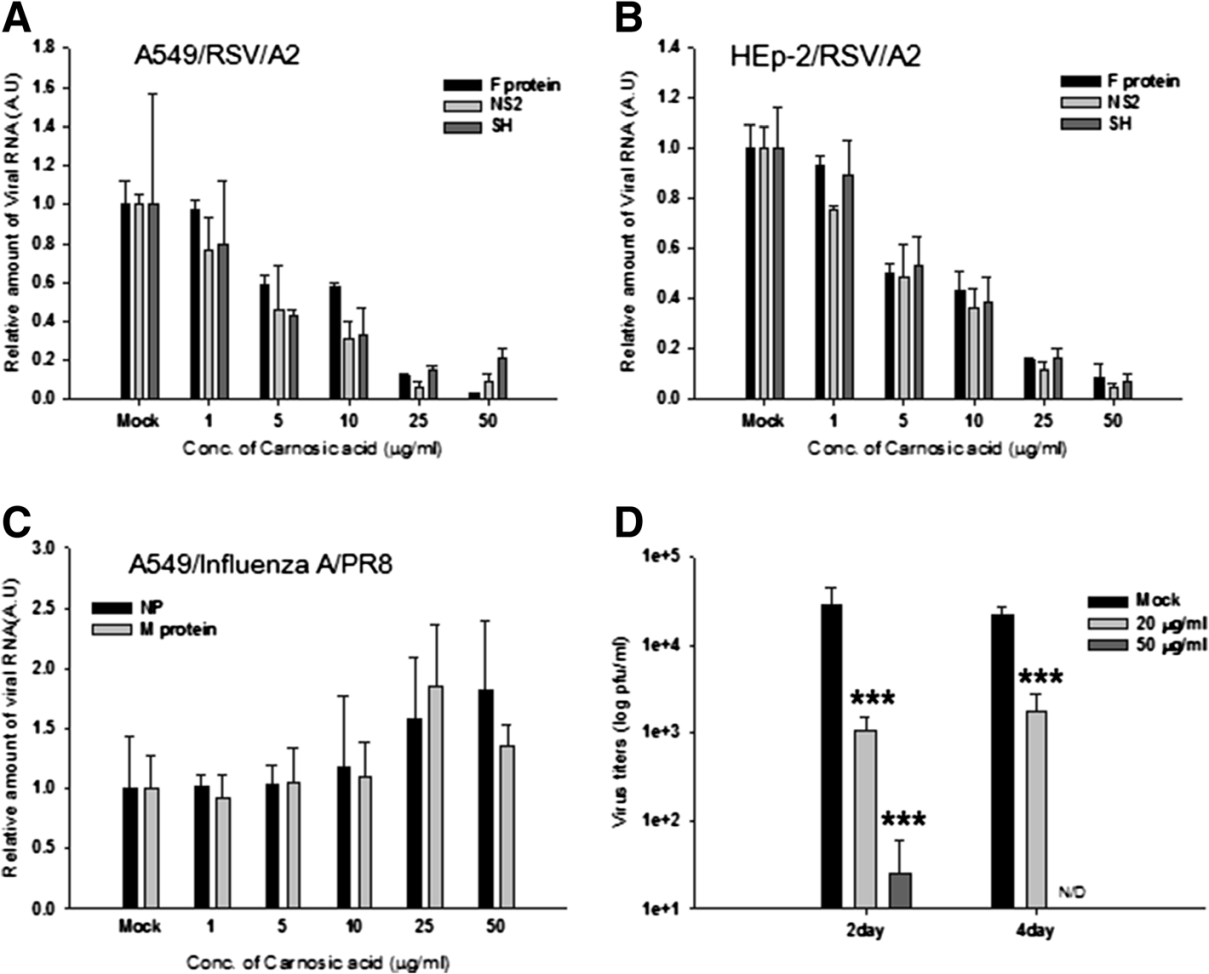
**Analysis of the anti-hRSV activity of carnosic acid.** A549 cells **(A)** and HEp-2 cells **(B)** were treated with different concentrations of carnosic acid. Cells were infected with hRSV A2 virus (MOI 0.5) for 48 hours, followed by RNA preparation and RT-qPCR. The relative amount of RNA for hRSV F, NS2 and SH protein were determined by RT-qPCR. Data represent means ± SD. ** *p* < 0.01 versus Mock-treated (Mock). A.U.: Arbitrary unit. **(C)** The effect of carnosic acid on influenza A virus replication was tested in A549 cells in a similar manner. Cells were infected with influenza A/PR8 virus (MOI 0.01) for 24 hours and the relative amount of RNA for influenza A virus NP and P protein were determined by RT-qPCR. A.U.: Arbitrary unit. **(D)** Inhibition of progeny virus production by carnosic acid. A549 cells were treated with the indicated compounds 1 hour prior to hRSV infection (MOI 0.1). Two and four days after infection, progeny viruses were harvested and titrated by plaque assay. All the experiments were performed in triplicate and data represent number of plaques (means ± SD). N/D.: not detected. *** *p* < 0.001 versus Mock-treated (Mock).

**Figure 7:**
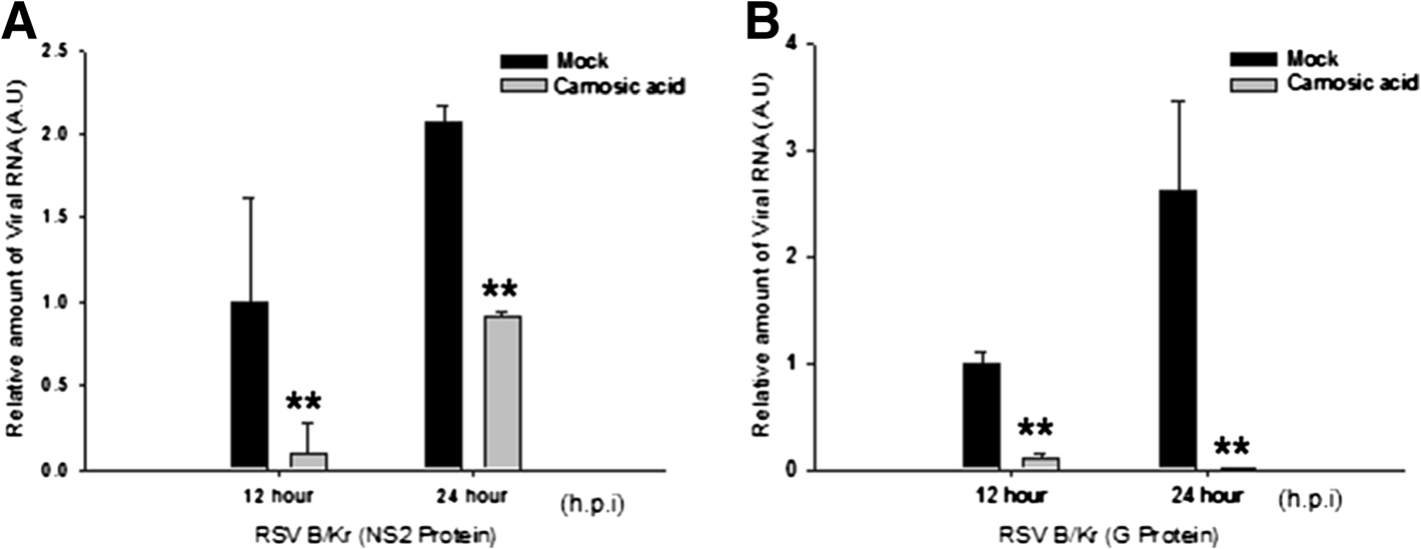
**Inhibition of both A- and B-type hRSV by carnosic acid.** Effects of carnosic acid on the synthesis of viral proteins from hRSV B/KR virus were analyzed by RT-qPCR. A549 cells were treated with carnosic acid 1 hour prior to infection. Twelve and twenty-four hours after virus infection, RNA levels of each viral protein were quantified by RT-qPCR using type-specific primers. NS2-specific primers **(A)** and G protein-specific primers **(B)** are used to assay antiviral activity against hRSV B/KR. All the experiments were performed in triplicate and data represent means ± SD. ** *p* < 0.01 versus Mock-treated (Mock). A.U.: Arbitrary unit.

In experiments using A549 cells and HEp-2 cells, transcription of F, NS2 and SH proteins of hRSV A2 was reduced by treatment with carnosic acid in a concentration-dependent manner (Figure 6A, B). Estimated average half-maximal inhibitory concentration (IC_50_) values of carnosic acid for RNA synthesis of viral proteins (F, NS2, and SH protein) in A549 cells and HEp-2 cells were 19.58 μM (6.51 μg/ml) and 20.19 μM (6.71 μg/ml), respectively. Effect of carnosic acid on the replication of influenza virus A was tested to examine whether antiviral activity of carnosic acid is hRSV specific or not. Unlike hRSV, viral RNA synthesis from influenza A virus infected A549 cells was not inhibited by the treatment of carnosic acid, indicating that carnosic acid specifically inhibits hRSV replication (Figure 6C). In addition, this result support that antiviral activity of carnosic acid is not due to the nonspecific effect on host cells. The anti-hRSV activity of carnosic acid was further confirmed by virus yield-reduction assay. Two days after infection, treatment with 20 μg/ml (60 μM) or 50 μg/ml (150 μM) of carnosic acid reduced virus progeny production by 26-fold and 1160-fold, respectively (Figure 6D). No viruses were detected in cells treated with 50 μg/ml (150 μM) of carnosic acid 4 days after infection (Figure 6D), confirming the inhibitory activity of carnosic acid against hRSV. Carnosic acid also significantly inhibited NS2 and G protein RNA synthesis of a hRSV B-type clinical isolate (hRSV B/KR), indicating that carnosic acid can suppress both A- and B-type hRSV (Figure 7A and B).

### Inhibition of hRSV replication and infection by carnosic acid

**Figure 8:**
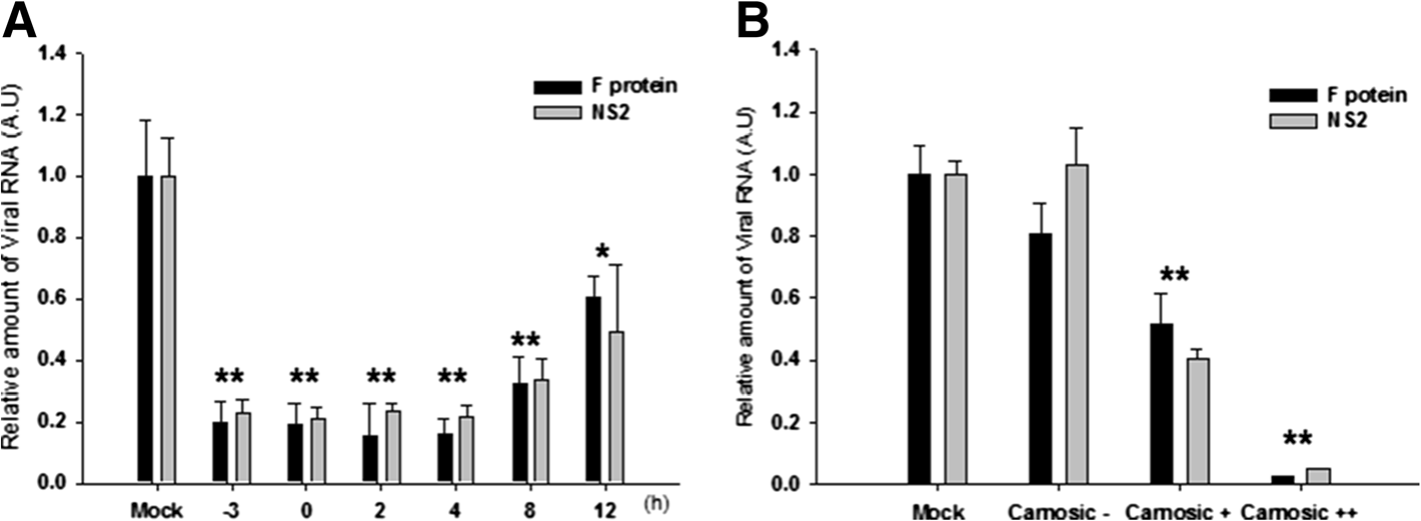
**Inhibition of hRSV replication and infection by carnosic acid. (A)** A549 cells were treated with carnosic acid at various times before and after virus inoculation. Inhibitory effects were analyzed by measuring the synthesis of RNA for viral F and NS2 proteins. **(B)** Cells were treated with carnosic acid for 1 hour. After washing five times with PBS to remove carnosic acid, cells were infected with hRSV A2 for 1 hour as follows: Car-, cells infected with hRSV without carnosic acid; Car+, cells infected with hRSV inoculum containing carnosic acid, then washed with PBS before further incubation; Car++, cells infected with hRSV inoculum containing carnosic acid and then further incubated in carnosic acid-containing media. Forty-eight hours after infection, viral protein synthesis was analyzed by RT-qPCR. All the experiments were performed in triplicate and data represent means ± SD. * *p* < 0.05 versus Mock-treated (Mock). ** *p* < 0.01 versus Mock-treated (Mock). A.U.: Arbitrary unit.

To understand the mechanism of hRSV inhibition by carnosic acid, we investigated the kinetics of carnosic acid-mediated inhibition of hRSV RNA synthesis. Pretreatment of cells with carnosic acid 3 hours prior to infection inhibited hRSV RNA synthesis by approximately 80%, based on quantitative determination of RNA for F and NS2 protein. Treatment with carnosic acid 4 hours after infection inhibited synthesis of RNA for F and NS2 protein to a similar degree (~80%) as pretreatment (Figure 8A). Post-treatment with carnosic acid 8 and 12 hours after infection still inhibited F protein RNA synthesis by about 70% and 50%, respectively (Figure 8A). Although these results suggest that carnosic acid inhibits hRSV replication, we also tested whether carnosic acid affected the initial infection of hRSV. We first treated A549 cells with 10 μg/ml of carnosic acid for 1 hour. Cells were then infected with hRSV for 1 hour and, after extensive washing with PBS, were further incubated with fresh medium with or without carnosic acid. In accord with our previous data, hRSV RNA synthesis in cells cultured in medium containing carnosic acid (Car++) was less than 10% of that in cells treated with vehicle (Car-). Moreover, treatment with carnosic acid only during the infection process (Car+) resulted in a 50% reduction of hRSV RNA synthesis, indicating that carnosic acid may also inhibit hRSV infection (Figure 8B). Viral RNA synthesis in cells infected with hRSV without carnosic acid (Car-) was similar to that in cells that had never been treated with carnosic acid (Con), confirming that the inhibition is not due to a change in cell physiology, such as altered interferon production or receptor expression.

In this study, we have shown that carnosic acid effectively suppresses hRSV replication. Carnosic acid showed similar suppressive effect against both A- and B-type hRSV, while it did not inhibit influenza A virus replication. Since carnosic acid did not induce significant cell death and interferon production at the used concentration, it is not likely that the inhibitory effect is due to the effect on host cells. Failure to suppress influenza A virus also support that notion. Moreover, carnosol, which has similar structure and possesses antioxidative activity, was unable to inhibit hRSV RNA synthesis. It suggests that the suppressive effect of carnosic acid is not due to the antioxidative activity of carnosic acid. Although the mechanism by which carnosic acid inhibit the replication of hRSV is unclear, it is likely that it affect viral factors such as viral proteins. In our study, we have shown that the levels of viral RNAs were significantly lowered by the treatment of carnosic acid on infected cells by using primers complementary to F, SH, NS2 and G proteins. Since carnosic acid is unable to inhibit the replication of influenza A virus, which also use negative sense ssRNA as their genome, it is not likely carnosic acid inhibits hRSV by directly interacting with viral RNAs. Given that carnosic acid inhibits hRSV replication by affecting viral proteins, one possible mechanism is carnosic acid act as an inhibitor of viral RNA synthesis by affecting viral proteins, which are related to viral RNA synthesis. For example, carnosic acid may directly affect RNA polymerase activity of hRSV L protein or cofactor P protein to suppress hRSV RNA synthesis. Targeting M2 protein by carnosic acid also can be a possible hypothesis, since M2 protein of hRSV has been known to be important for efficient transcription of viral mRNAs [30]. Time-of-addition study results suggest that carnosic acid also affect the initial infection step. Thus, it can be postulated that carnosic acid may play an additional different role in inhibiting hRSV by interacting viral surface proteins such as F, G and SH proteins. Further investigations such as isolation of resistant mutant viruses or identification of viral proteins which interact with carnosic acid will reveal the more specific mechanism of inhibition.

## Conclusion

The use of ribavirin for the treatment for hRSV infection is limited by its toxicity and limited efficacy; thus, there is an urgent need for the development of new therapeutic agents against hRSV. The current study demonstrates that carnosic acid, which has been used as a preservative and antioxidant in food, effectively inhibits the replication of hRSV. Carnosic acid not only reduced viral RNA synthesis, it also inhibited the initial infection of hRSV. Consequently, hRSV progeny virus production was greatly reduced by carnosic acid treatment. Since treatment with carnosic acid both pre- and post-exposure suppressed the replication of hRSV, this compound might be a potential prophylactic and therapeutic agent against hRSV infection.

## Material and methods

### Cells and viruses

The human larynx carcinoma cell line HEp-2 (CCL-23) and human adenocarcinoma alveolar basal epithelial cell line A549 were maintained in Dulbecco’s modified Eagle’s medium (DMEM) containing 10% fetal bovine serum (FBS) and penicillin/streptomycin (100 U/mL). hRSV A2 and hRSV KR/B (subgroup B) were described elsewhere [31]. Viruses were propagated by infecting HEp-2 cell monolayers with a previously prepared small-scale isolate stock at a multiplicity of infection (MOI) of 0.01. Viruses were harvested when cytopathic effects were greater than 60% and were titrated by plaque assay as described previously [32]. Influenza A/Puerto Rico/8/1934 virus was propagated in specific pathogen-free embryonated eggs as described previously [33].

### Plant materials

The rosemary plant (*Rosmarinus officinalis* L.) and other plants used in this study were purchased from a local market at Seoul. A voucher specimen (No. 2012-ROOF01) has been deposited in the Laboratory of Natural Product Medicine, College of Pharmacy, Kyung Hee University.

### Screening of plant extracts for anti-hRSV activity by microneutralization assay

Plant extracts were initially screened using microneutralization assays, as described previously [34]. Briefly, methanol (MeOH) extracts of each plant were dissolved in DMSO and serially diluted with Phosphate buffered saline (PBS). MeOH extracts (20 μg/ml) of each plant were added to HEp-2 cells (5 **×** 10^4^ cells/well) together with 1000 plaque-forming units (pfu) of hRSV A2 virus. Final concentration of DMSO was 2% in all samples. Three days after infection, cells were fixed with 3.7% formaldehyde. After blocking with 5% skim milk, the expression of hRSV fusion (F) protein was assessed by enzyme-linked immune sorbent assay (ELISA) using mouse anti-F monoclonal antibody (Sino Biological) and horseradish peroxidase (HRP)-conjugated anti-mouse IgG.

### Extraction and isolation of compounds

Air-dried, powdered aerial parts of *R. officinalis* (12.5 g) were extracted three times with 70% ethanol (EtOH) by maceration. The extracts were combined and concentrated *in vacuo* at 40°C to give a 70% EtOH extract (3.11 g). The extract was suspended in H_2_O (100 mL) and successively extracted with *n-*hexane (3 × 100 mL), ethyl acetate (EtOAc; 3 × 100 mL), and butanol (BuOH; 3 × 100 mL) to give *n-*hexane-soluble (719.7 mg), EtOAc-soluble (950.2 mg), BuOH-soluble (460.6 mg), and water-soluble extracts (966.5 mg), respectively. Based on preliminary biological test results, the EtOAc-soluble extract (940 mg) was subjected to column chromatography on silica gel to obtain pure compounds. The purities (>95%) and structures of the obtained compounds were determined by high-performance liquid chromatography (HPLC) and nuclear magnetic resonance (NMR) spectroscopy. The structures of the isolates were identified based on comparisons of physical and spectroscopic data with published values.

### RNA isolation and reverse-transcription quantitative real-time polymerase chain reaction (RT-qPCR)

Expression of viral genes was analyzed by RT-qPCR using CFX-9000 (Bio-Rad) real-time PCR. Total RNA was extracted from mock-infected or hRSV-infected cells using an RNeasy RNA extraction kit (Qiagen), according to manufacturer’s instructions. cDNA was synthesized from 1 μg of total RNA using Superscript III reverse transcriptase (Invitrogen) with oligo_20_(dT) primers. Two microliters of synthesized cDNA was used as a template for quantitative PCR. Sequences of primers used for this study are listed in Table 1.

**Table 1:** List of primers used for RT-qPCR in this study

**Target RNA**	**Sense (5′-3′)**	**Anti-sense**
hRSV A2 F	CCACAATCCTCGCTGCAGTC	GGCTCCTAGAGATGTGATAACGG
hRSV A2 NS2	ATTGGCATTAAGCCTACAAAGCA	CTTGACTTTGCTAAGAGCCATCT
hRSV A2 SH	AATTGGAAGCACACAGCTAC	TTGCATTTGCCCCAATGTT
hRSV B G	ATGATTGCAATACTAAA	ACACTGGTATACCAACC
Influenza A NP	TGTGTATGGACCTGCCGTAGC	CCATCCACACCAGTTGACTCTTG
Influenza A M2/M1	CTTCTAACCGAGGTCGAAACGTA	GGTGACAGGATTGGTCTTGTCTTTA
Interferon-β	GAACTTTGACATCCCTGAGGAGATT	TGCGGCGTCCTCCTTCT
Interferon-λ1 (IL-29)	GGACGCCTTGGAAGAGTCAC	AGCTGGGAGAGGATGTGGT
Interferon-λ2 (IL-28)	AGGGCCAAAGATGCCTTAGA	TCCAGAACCTTCAGCGTCAG
β-actin	TGCCGCATCCTCTTCCTC	CGCCTTCACCGTTCCAGT

### Effect of carnosic acid on interferon production upon hRSV infection

The levels of interferon mRNAs were determined by RT-qPCR. A549 cells were treated with 20 μg/ml of designated compound one hour prior to hRSV infection. Cells were then incubated for additional 6 hours, followed by RNA isolation and RT-qPCR. To analyze the effect of compounds on interferon production without viruses, cells were incubated with vehicle or designated compounds (20 μg/ml) for 6 hours without virus infection. Primers complementary to interferon-β, λ1 and λ2 (Table 1) were used to analyze mRNA levels. mRNA levels were normalized to β-actin mRNA levels.

### Effect of carnosic acid on viral RNA synthesis

A549 cells were treated with different concentrations of designated compound one hour prior to hRSV infection. Cells were then infected with hRSV A2 (MOI 0.5) for an hour. After washing to remove unbound viruses, cells were further incubated for 48 hours in media containing the same concentrations of each compound. Total RNAs were isolated and used for RT-qPCR. The replication of hRSV A2 virus was analyzed using primers complementary to F protein, SH protein and non-structural protein 1 (NS1). To determine the replication of hRSV B virus, primers complementary to NS2 and G protein of hRSV B type were used for quantitative PCR. The replication of influenza A virus was analyzed using primers complementary to NP protein and M2/M1 protein. Viral RNA levels were normalized to β-actin mRNA levels. Sequences of primers used are listed in Table 1.

### Cytotoxicity assay

Cell viability was determined by a MTT (3-(4,5)-dimenthylthiahiazo(−z-y1)-di-phenytetrazoliumromide) cell viability assay. A549 cells in a 96-well plate were cultured in DMEM containing increasing concentrations of carnosic acid for 48 h. Next, the culture medium was replaced with fresh medium containing 20 μl of MTT (5 mg/ml) for 4 h. After that the MTT containing medium was aspirated and 200 μl of DMSO was added to lyse the cells and solubilize the water insoluble formazone. Absorbance of the lysates was determined on a microplate reader at 570 nm.

### Apoptosis assay

Carnosic acid-induced apoptosis was determined by annexin V and PI double labeling. Cells treated with or without carnosic acid for 24 or 48 hours were washed with PBS, and stained with 5 μl of FITC-conjugated annexin V and 5 μl of PI (50 μg/ml) in 100 μl binding buffer (10 mM HEPES at pH 7.4, 140 mM NaCl, and 2.5 mM CaCl_2_). The fluorescence of annexin V and PI were monitored by FC500 fluorescence-activated cell sorting (FACS) cater-plus flow cytometry (Beckman Coulter, CA, USA) at an excitation wavelength of 488 nm and emission wavelengths of 525 and 625 nm, respectively. Five thousand events were collected per sample.

### Virus yield-reduction assay

A549 cells were treated with designated extracts (20 μg/ml) 1 hour prior to hRSV inoculation. hRSV (MOI 0.5) was then added and cells were incubated for 1 hour to permit viral internalization. Free virus particles were removed by washing three times with phosphate-buffered saline (PBS), and infected cells were further incubated in medium containing 2% FBS and vehicle (DMSO) or 20 μg/ml of designated extracts. Viruses were harvested 3 days after infection and subsequently titrated by plaque assay. To determine the effect of carnosic acid, carnosic acid (20 μg/ml) was added to cells instead of extracts. Viruses were harvested 2 or 4 days after infection and subjected to plaque assay. Viral titer was expressed as pfu/mL.

### Time-of-addition study

A549 cells were seeded and incubated overnight. Cells were treated with carnosic acid (10 μg/ml) at different time points before and after viral inoculation (MOI 0.5). Total RNA was prepared from cells 48 hours after viral inoculation, followed by RT-qPCR using primers specific for F and NS2 proteins. The effect of carnosic acid on the initial infection by hRSV was examined by treating cells with carnosic acid 1 hour prior to inoculation with virus. After inoculation, cells were incubated for 1 hour to allow viral internalization. Free virus and carnosic acid were removed by extensive washing with PBS. As a control, cells were treated with carnosic acid for an hour and washed before viral inoculation to exclude the possibility that carnosic acid altered viral infection or replication by affecting cell physiology. Total RNA was prepared from cells 48 hours after viral inoculation and subjected to RT-qPCR using primers specific for F and NS2 proteins, as described above.

### Statistical analysis

Microneutralization assays and RT-qPCR assays were repeated at least three times. Values were expressed as means and standard deviation from triplicate samples of a representative result. Statistical comparisons between the control and treated groups were analyzed using the Student’s *t*-test. The level of statistical significance was set at either P<0.05 (*), 0.01 (**) or 0.001(***).

## Competing interests

The authors declare that they have no competing interests.

## Authors’ contributions

SHB, LNR, CJ, LKT and IKS designed and conceived the experiment. RB and JDS prepared the extracts, fractions and purified compounds. SHB, CMS, CHE and KHI participated in the studies on antiviral activity. SHB, CJ, JDS, LKT and IKS participated in data interpretation and wrote the manuscript. All authors read and approved the final manuscript.
